# A scoping review of spinal cord stimulation for phantom limb pain

**DOI:** 10.1016/j.inpm.2025.100571

**Published:** 2025-03-12

**Authors:** Stephen Jaffee, Rhea Verma, Mariam Vaezi, Trent Kite, Nestor Tomycz

**Affiliations:** a Department of Neurosurgery, Allegheny Health Network Neuroscience Institute, Pittsburgh, PA, USA; b Drexel University College of Medicine, Philadelphia, PA, USA

**Keywords:** Phantom limb pain, Spinal cord stimualtor, SCS, Pain

## Abstract

**Background:**

Phantom limb pain (PLP) is a debilitating condition that affects individuals following limb amputation. While medical management with anticonvulsants and physical therapy is the first-line treatment, spinal cord stimulation (SCS) has emerged as an option for patients with persistent, refractory pain.

**Objectives:**

This study aimed to evaluate the current literature on SCS for PLP, focusing on pain reduction and associated complications.

**Methods:**

A systematic review was conducted on reports of adults (≥18 years) with phantom limb pain treated with spinal cord stimulation. Titles and abstracts were screened, followed by a full-text review based on predefined inclusion criteria. Extracted data included sample size, SCS lead placement, pain reduction, visual analog scale (VAS) and brief pain inventory (BPI) scores, and complications. Descriptive statistics were used for analysis.

**Results:**

Five reports met inclusion criteria, comprising 33 patients. Of these, 18 % of patients achieved 90–100 % pain reduction, 15 % had ≥80 % reduction, 6 % had ≥60 % reduction, and 15 % experienced ≥50 % reduction. Post-SCS visual analog scores were reported in two studies; one study reported a mean 50 % reduction in visual analog scale scores (VAS), while another found a median brief pain inventory (BPI) reduction of 43.5 %. Complications across all 33 patients included wound infection (6 %), transient weakness (3 %), cerebrospinal fluid leak (3 %), allergic dermatitis (3 %), and electrode site cyst (3 %). Most patients (84.8 %) received epidural lead placement, while 15.2 % had subdural placement.

**Conclusions:**

SCS may be effective in reducing pain in some PLP patients.However complications exist, with wound infection being the most common complication. The mechanism of action remains unclear, but PLP likely involves both central and peripheral pathology, which complicates treatment. Historically, SCS has shifted from subdural to epidural lead placement to minimize complications, with recent reports exploring dorsal root ganglion stimulation for more targeted pain relief. Spinal cord stimulation appears to provide meaningful pain reduction for patients with phantom limb pain, with a subset achieving near-complete relief. However, reported outcomes vary and complications remain a concern. Given the limited number of reports and small sample sizes, further research is needed to assess long-term efficacy and to minimize complications.

## 1 Introduction

Phantom limb pain (PLP) is a chronic condition characterized by persistent pain following amputation of a limb. The estimated incidence following limb amputation is 50–80 % [1]. Presentation of this phenomenon is highly variable. Classical descriptions include paroxysmal shocking sensations, stabbing, burning, and throbbing pain [1]. Contemporary management is either with pharmacological or surgical approaches [1]. Medical therapy is primarily with anticonvulsants while surgical management is much more heterogenous [1]. Previous literature has described surgical management of PLP with stump revision, neurectomy, rhizotomy, sympathectomy, cortical stimulation, and spinal cord stimulation (SCS) [1,2].

SCS is an FDA approved therapy for PLP which has gained traction over the past few years [1]. SCS functions by transmitting persistent electrical signals to the dorsal column of the spinal cord either through subdural or epidural electrodes [3,4]. Typically, this therapy is reserved for individuals with moderate pain, for a duration of at least 6 months with reduced quality of life (QoL) who have previously tried and failed medical therapy and less invasive management options [1]. While limited, there has been published literature with promising results with respect to pain reduction [1,3,5,6]. However, there are associated complications such as wound infection, incision site pain, lead migration, nerve root/spinal cord injury and cerebrospinal fluid leak (CSF) [1]. Long term outcomes with respect to pain reduction efficacy and complication rates are lacking.

We sought to provide an overview of the current literature as it relates to SCS as a therapeutic modality for PLP. Previous systematic reviews on this topic have included mixed cohorts of chronic pain patients with various underlying causes. Since phantom limb pain (PLP) has distinct pathophysiological features, we focused our analysis on studies specifically addressing this patient population, rather than broader chronic pain cohorts. We have highlighted key components of contemporary studies and case reports and have presented and contextualized their results. Furthermore, where appropriate, we have analyzed this data and provided aggregate level outcomes focusing on pain reduction and complications.

## 2 Methods

### 2.1 Literature search

**Fig. 1:**
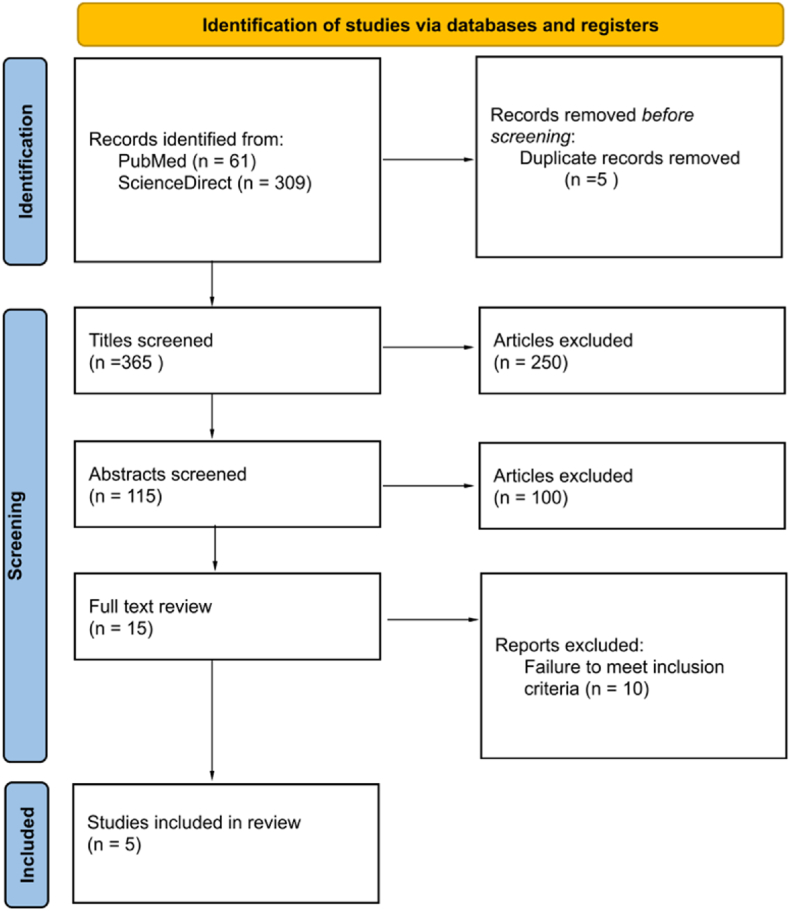
Diagrammatic representation of literature search in accordance with PRISMA 2020.

A systematic review was conducted in accordance with the Preferred Items in Systematic Reviews and Meta-analysis (PRISMA) (Fig. 1). The inclusion criteria for our review were as follows: (1) Patient's ≥ 18 years of age, (2) diagnosis of PLP, (3) management with SCS (4) articles with a primary focus on outcomes of PLP patients treated with SCS. Given the limited literature available on the topic, we expanded the range of acceptable article types from formal studies to case reports and cases series, we have therefore referred to these collectively as “reports” throughout the study. A PubMed and Science Direct database search was conducted using the following combination of search phrases connected by Boolean operators: (“Phantom Limb Pain” OR “PLP”) AND (“Spinal cord stimulation” or “SCS”). No date or language filters were applied to the search. Search results were initially filtered by title on the basis of relevance to the keywords used in the search. The remaining article's abstracts were screened for relevance. Finally, the remaining articles underwent full text review and were selected based on meeting the pre-defined inclusion criteria. In an effort to augment our results we have included two cases from our own institution in the review. These cases have not previously been published. Two independent authors conducted the search (T.K) and (S.J), while a third (R.V) mediated any discrepancies.

### 2.2 Data extraction

Two independent authors (T.K) and (S.J) extracted data from the reports deemed suitable for inclusion in the final analysis. The following pre-determined data were collected: number of subjects in each study, SCS lead placement, percent pain reduction post-procedurally, post-procedure visual analog scale (VAS) and brief pain inventory (BPI), and procedure related complications. All data were collected in a centralized database saved for further analysis.

### 2.3 Quality assessment

A Joanna Briggs Institute (JBI) checklist for case series and reports was utilized to assess article suitability for inclusion in the final analysis. Articles with a score of ≥6 were deemed to be “good” quality and suitable for inclusion in the final analysis.

### 2.4 Statistical analysis

Continuous and categorical variables were described using descriptive statistics (median (IQR), frequency (%)) where appropriate. All calculations were performed in GraphPad Prism version 10.3.1.

## 3 Results

Overall, 370 articles resulted from the PubMed and ScienceDirect search. Five reports were excluded as duplicates, leaving 365 reports for title review. After screening report titles for relevance to the search keywords, 250 articles were excluded leaving 115 reports for abstract review. After abstract review 100 reports were excluded with 15 reports remaining. From the final 15 reports the remaining 10 were excluded for failure to meet inclusion criteria. More specifically these 10 reports were excluded as they did not provide a primary analysis or an adequate subgroup analysis of patient outcomes for those undergoing SCS for PLP. Five total studies were selected for final analysis and all 5 (100 %) met the JBI threshold for inclusion in the final analysis. A representative PRISMA flow chart with these results is presented in Fig. 1 [12]. Overall, our final analysis included 33 patients, over 5 previously published reports and our cohort. Of these, 6 patients (18 %) had a 90–100 % improvement in pain rating following SCS, 5 patients (15 %) had ≥80 % pain reduction, 2 patients (6 %) had 60 % pain reduction, and 5 (15 %) of patients had at least a 50 % pain reduction. One report(16.7 %) reported post-SCS visual analog scale (VAS) scores reporting a mean 50 % reduction from pre-SCS baseline. Another report (16.7 %) reported brief pain inventory (BPI) outcomes post-SCS with a median BPI score of 9 (IQR: 4.3–13.8) which correlates to a 43.5 % reduction (IQR: 29.3–63.8 %) from pre-SCS baseline. With respect to post-SCS complications: wound infection was the most common 2 (6 %), followed by transient weakness 1 (3 %), CSF leak 1 (3 %), allergic dermatitis to implantable pulse generator 1 (3 %), and cyst at the electrode site 1 (3 %). Twenty-eight patients (84.8 %) had epidural lead placement and 5 (15.2 %) had subdural lead placement. A comprehensive summary of these results is tabulated in Table 1.

**Table 1:** A summary of key data presented across articles reporting PLP outcomes when treated with SCS.

Study	Patients (*N*)	Electrode System	Lead Location	Subjective Pain Relief	Post-SCS VAS	Change in baseline BPI following SCS	Post-SCS Complications
Rupesh et al. [1]	1	Dual channel dorsal column prime advance SCS system	C7 Cord (epidural)	50 %	4	NR	None
Nielson et al. [6]	5	NR	C3-4	(5/5) 100 %	NR	NR	Transient weakness
			T2-T3				CSF Leak
			T6-T7 (Subdural and Endodural)				Wound infection
Viswanath et al. [8]	4	Percutaneous octopolar system (Medtronic)	T10-T11	(4/4) >80 %	NR	13 (42 %)	Allergic dermatitis to implantable pulse generator
			T8-T9			14 (70 %)	Wound infection
			T10-T12			4 (25 %)	
			T12-L1 (Epidural)			5 (45 %)	
McAuley et al. [3]	12	Quadripolar paddle electrodes (Medtronic)	NR (Epidural)	1 (90 %)	50 % (mean reduction from pre-lead placement)	NR	Cyst at electrode site
				1 (80 %)			
				2 (60 %)			
				2 (50 %)			
				1 (0–25 %)			
				4 (NR)			
Miles and Lipton [5]	9	NR	Dorsal Column (undefined levels)	6 (excellent)	NR	NR	NR
			(Epidural)	1 (some)			
				2 (none)			
This study	2	Penta paddle lead (Abbott)	T9	1 (51–75 %)	NR	NR	None
			T9 (Epidural)	1 (>50 %)			
Abbreviations: SCS: spinal cord stimulation, VAS: visual analog scale, BPI: brief pain inventory, NR: not reported							

## 4 Discussion

### 4.1 Summary of findings

Overall, we found that most patients reported a post-SCS pain reduction of at least 50 %. Further, the most common complication was wound infection. SCS leads were more commonly placed epidurally versus subdurally. Confirming the heterogeneity of reports, we found that out of all the studies in the final analysis only 1 reported a BPI and VAS (standardized pain rating scales) following SCS lead placement. Out of the 10 patients in which spinal level stimulator placement was reported 2 (20 %) and 8 (80 %) were placed in the cervical and thoracic spine respectively. Five (83.3 %) studies exclusively placed leads epidurally, which is consistent to the switch to this approach documented after the 1970s. This review is one of the few existing pieces of literature isolating cohorts of patients with PLP treated with SCS and reporting on their outcomes.

### 4.2 Mechanism of SCS in PLP

The exact mechanism by which SCS effectively controls the symptomatology of PLP is currently undefined. Nonetheless PLP is generally considered to arise secondary to both central and peripheral pathology [6]. While the initial pathology is peripheral in origin, attempts at exclusively treating peripheral nerves have been unsuccessful at completely abolishing the phantom sensation [6]. Because of this, PLP is a subtype of central pain syndromes, which have been demonstrated to be under the influence of two primary pathways, the spinothalamic tract and multisystem afferent system (MAS) respectively [6]. The opposing dynamics of these systems produce the net effect of conscious perception of pain [6]. Electrophysiologic data presented by Noordenbos in the 1950s demonstrated the tight regulation between excitatory and inhibitory inputs across these pathways [1,7]. Derived from this work was the “Gate theory” of pain control, which is a widely accepted theory explaining the role of SCS in PLP [1,7]. This theory posits that dorsal column stimulation activates Aα fibers which in turn induce inhibition of transmissible pain signals to the cortex [1,2,7]. The lack of certainty surrounding the exact pathophysiology of PLP represents a primary challenge to implementing effective management. More precisely defined spinal cord targets and a better understanding of the electrophysiologic effects of stimulation would enable greater efficacy relative to the general non-specificity of contemporary SCS.

### 4.3 History of SCS for phantom pain

SCS for phantom pain was first introduced in the literature in the 1970s [5,6,[8], [9], [10],^13^,^14^]. Early approaches incorporated both subdural and endodural stimulation electrode placement [5,6,[8], [9], [10]]. To our knowledge Nielson et al., in 1975 reported the first case series describing SCS in the management of PLP [6]. Shortly after, in 1975 and 1978 respectively Hunt et al. and Miles and Lipton et al. published their series of PLP patients managed with SCS [5,10]. Miles and Lipton et al. demonstrated the potential for durable relief from phantom sensation upon follow up of patients years later noting that most of these individuals either no longer needed their stimulation or had markedly decreased usage [5]. A primary change in the approach to SCS involved the transition of subdural lead placement to primarily epidurally place leads in the 1980s and beyond [1]. Utilizing this approach likely decreases the risk of cerebrospinal fluid leak (CSF) with associated symptoms (postural headache and meningitis). In more recent reports the dorsal root ganglion (DRG) has been increasingly utilized as a lead placement site [1,7,8]. Briefly the benefits of DRG stimulation over SCS are the ability to limit extraneous stimulation of nerves in non-painful sites, decrease loss of efficacy secondary to a patient's postural changes, and improved quality of life [11]. While early reports are promising, further work in the form of prospective clinical trials is warranted.

### 4.4 Subjective pain relief

McAuley et al. described in depth a few factors which may predict the success of SCS in PLP [3]. The authors described that there may be a relationship between the duration of pain and the extent to which alternative therapies have been attempted prior to SCS lead placement [3]. This represents a reasonable theory as this patient population may be largely composed of highly treatment resistant pathology subtypes [3]. Perhaps earlier intervention with SCS may produce better outcomes. They also highlight that the mental health status of patients has the potential to interact with SCS success, as high anxiety and depressive states have been noted to influence the success of neuromodulation previously [3]. The brief pain inventory (BPI) and visual analog scale (VAS) are standardized pain assessments which can feasibly be implemented in the clinical workflow. Unfortunately, many of the studies analyzed in this paper did not report on these outcomes. In an effort to increase the consistency and generalizability of future work, utilizing standard metrics by which to judge the success of a given therapy will be important. One such outcome which has become commonplace is monitoring the frequency and extent of opioid pain medication use in these patients. Adjusting pain response for opioid induced analgesia is extremely important as medical practice has evolved to limit the use of this class of medication recently.

### 4.5 Complications

As with any procedure involving the spinal cord there exists a risk of CSF leak, typically occurring via inadvertent puncture of the dura with SCS implantation. Among the most serious complications is nerve root or spinal cord injury, however this is a rare event. Eleven of the twelve patients in the cohort described by McAuley et al. experienced technical issue with migrating lead position and incorrect stimulation sites [3]. While this is an issue not specific to PLP patients, it underscores the importance of correct and secured lead placement such that maximal stimulation-based benefits can be derived. Improved outcomes over time may be seen from refinements in SCS technology alone. While this may be the case in a proportion of patients, one cannot exclude the fact that in some patients' fundamental changes in neural circuitry may preclude effective stimulation [3].

### 4.6 Limitations and future directions

The reports included in this review are limited by small sample sizes and heterogenous study designs. Further, many of the reports fail to discuss in depth the SCS parameters used in these studies. Next steps involve further refinement of pathophysiology of PLP and the interaction between SCS and neurotransmission associated with PLP. Additionally, refining spinal cord targets, especially given the rise of DRG stimulation will be important. Further emphasis on reporting data on pre-SCS treatment strategies, pre-SCS pain duration, and neuropsychological profiles of patients may yield valuable insights as these have been postulated to predict response in this patient population. As SCS is limited by a relatively non-specific targeting approach it is critical to track associated paresthesia in the targeted dermatomal region. This is particularly important as pain outcomes may not achieve clinical significance in a direct comparison with DRG stimulation, high-frequency, or burst stimulation. Therefore, understanding associated effects will assist in clinical decision making Additionally, as the role of alternative techniques of SCS like high frequency and burst evolve, there are additional opportunities to improve upon the efficacy of SCS. To date, there is unclear evidence to suggest the superiority of these aforementioned SCS techniques over conventional approaches. Much of the data is mixed with respect to pain outcomes. Furthermore, a major limitation of the previous reports is a lack of neurostimulation data. Future trials need to focus on meticulously collecting this data for a comparison across a range of stimulation modalities. All in all these emerging techniques may prove useful, however significant research is needed to adequately discuss the merits of these techniques in the context of PLP.

## 5 Conclusion

Based on the existing literature, SCS has the potential to provide excellent pain relief in patients with PLP. However, the question remains whether the pain relief achieved in this cohort is transient versus durable. Longer term follow up on future cohorts will be necessary to determine this. Additionally, while the rate of medical complications is not concerning, there are still nuances related to SCS lead placement that potentially impact outcomes across each patient. Finally, delineating a suitable target for stimulation will be a crucial research investigation spanning the next few decades. As more evidence accumulates regarding the role of DRG versus SCS in the neuromodulation literature we can expect to see comparisons of PLP outcomes with respect to both of these targets. As data is reported it will be critical to use standardized pain assessments to judge their efficacy across a variety of studies.

## Declaration of competing interest

The authors declare that they have no relevant conflicts of interest to disclose.
